# Immunomodulatory Strategies Targeting Dendritic Cells to Improve Corneal Graft Survival

**DOI:** 10.3390/jcm9051280

**Published:** 2020-04-28

**Authors:** Alfrun Schönberg, Matthias Hamdorf, Felix Bock

**Affiliations:** 1 Department of Ophthalmology, Faculty of Medicine and University Hospital Cologne, University of Cologne, 50937 Cologne, Germany; alfrun.schoenberg@uk-koeln.de (A.S.); matthias.hamdorf@uk-koeln.de (M.H.); 2 Center for Molecular Medicine Cologne (CMMC), University of Cologne, 50937 Cologne, Germany

**Keywords:** cornea transplantation, graft rejection, immunomodulation, tolDCs, Tregs, hemangiogenesis, lymphangiogenesis, tolerance

## Abstract

Even though the cornea is regarded as an immune-privileged tissue, transplantation always comes with the risk of rejection due to mismatches between donor and recipient. It is common sense that an alternative to corticosteroids as the current gold standard for treatment of corneal transplantation is needed. Since blood and lymphatic vessels have been identified as a severe risk factor for corneal allograft survival, much research has focused on vessel regression or inhibition of hem- and lymphangiogenesis in general. However, lymphatic vessels have been identified as required for the inflammation’s resolution. Therefore, targeting other players of corneal engraftment could reveal new therapeutic strategies. The establishment of a tolerogenic microenvironment at the graft site would leave the recipient with the ability to manage pathogenic conditions independent from transplantation. Dendritic cells (DCs) as the central player of the immune system represent a target that allows the induction of tolerogenic mechanisms by many different strategies. These strategies are reviewed in this article with regard to their success in corneal transplantation.

## 1. Introduction

The cornea is the most transplanted tissue worldwide. Introduction of a corneal allograft in an avascular host bed is considered as highly successful with survival rates of about 90% after 1 year, and 55% after 15 years. These favorable outcomes are attributed to the limitation and modulation of responses towards inflammatory stimuli—regarded as immune privilege—as well as to the active maintenance of the cornea’s avascular status—regarded as angiogenic privilege [1]. Avascularity is attributed to the presence of antiangiogenic and immune modulatory factors [2,3,4,5,6] in both the aqueous humour as well as in the cornea itself [7].

However, breakdown of these privileges by severe inflammatory stimuli leads to the host bed’s pre-vascularization, consequently diminishing the 1-year survival rate to 50% or even lesser. Similar to other transplantations, immune-mediated rejections remain the most common cause for graft failure [8]. Therefore, corneal graft rejection is currently predominantly managed by the use of immunosuppressive agents such as corticosteroids. Both systemic as well as topical application have severe side-effects, including cataract, glaucoma, conjunctival necrosis, or impaired wound healing, emphasizing the need for alternative pharmacological strategies to promote graft survival [9,10].

One of the most important immune cell subsets for immune-mediated graft rejection are dendritic cells (DCs). In general, they are well known as central mediators of immune responses [11]. These bone-marrow derived, professional antigen-presenting cells (APCs) serve as a link between the innate and adaptive immune system. On the one hand, they efficiently stimulate B and T lymphocytes to fight infections and induce inflammation; on the other, they induce and maintain tolerance [11,12]. Immature DCs (iDCs) are characterized by low expression of major histocompatibility complex class II (MHCII) as well as of the co-stimulatory molecules CD80, CD86, and CD40 [13,14]. Moreover, they do not produce and secrete pro-inflammatory cytokines [14]. The cornea itself harbors a low amount of resident APCs, which are of immature phenotype [15,16,17,18]. Contact with an inflammatory stimulus, such as alloantigens, induces the maturation of DCs (mDCs), which is associated with an increased expression of MHCII and co-stimulatory molecules on the surface [12]. Furthermore, a third DC population has been characterized, which describes a phenotype in-between iDCs and mDCs. These tolerogenic DCs (tolDCs) express variable levels of MHCII, but only moderate amounts of CD80, CD86, and CD40 [13,14,19,20].

The DC phenotype critically determines the fate of T cell response which, in turn, is mainly responsible for the graft outcome. Full activation and expansion of T cells is considered as a two-step mechanism but is furthermore dependent on a third signal [21]. First, antigen presented by APCs in the context of MHC presentation is recognized via the T cell receptor (TCR), resulting in T cell activation [22]. Second, clonal expansion and differentiation of activated T cells rely on a co-stimulatory signal provided by different surface molecules on APCs and T cells [23]. Furthermore, the nature of secreted cytokines shapes the immune response [24]. While IL-12p70 is classified as a pro-inflammatory cytokine, transforming growth factor β (TGF-β) and IL-10 show anti-inflammatory properties. tolDCs present antigen to T cells as efficient as mDCs, but the low amount of co-stimulatory molecules is not efficient enough to trigger a pro-inflammatory T cell response [13,25]. Furthermore, they secrete low amounts of IL-12p70, but express elevated levels of TGF-β, IL-10, and indoleamine-2,3-dioxygenase (IDO) [19,20,25]. The tolDC–T cell interaction rather leads to induction of T cell anergy, clonal deletion, apoptosis of naïve, and memory T cells or, most interestingly, to the generation and expansion of regulatory T cells (Tregs) expressing CD4, CD25, and Foxp3 [19,25,26,27].

During transplantation, T cells can be activated via the direct or indirect pathway of allorecognition. In the direct response, recipient T cells recognize intact MHC molecules on the surface of donor APCs [28]. The indirect pathway, on the other hand, includes the processing of donor MHC molecules by recipient APCs, which in turn present the foreign peptide on their surface to T cells [28]. More recently, a third pathway has been described. The ‘semi-direct’ stimulation of recipient T cells involves donor-derived extracellular vesicles, such as exosomes, which carry donor MHC molecules on their surface. Those vesicles are taken up by recipient APCs, which then present the intact donor MHC molecules on their surface to T cells [28]. The interaction between DCs and T cells and subsequent induction of adaptive responses against foreign tissue after transplantation takes place in the draining lymph nodes (dLNs). It requires the transport of antigen-loaded DCs and soluble antigenic material to the dLNs, which is enabled by afferent lymphatic vessels [29]. Efferent blood vessels, in turn, allow the migration from activated T cells to the site of inflammation [30].

The ingrowth of blood and lymphatic vessels into the cornea therefore facilitates immune cell trafficking and promotes rejection of the transplant. Several studies showed that targeting (neo) vascularization of those vessels is effective in decreasing rejection rates and promising in terms of clinical application [31]. Therefore, research focusses on identifying factors involved in hem- and lymphangiogenesis, determining their role in this process, and the effect of their blocking or application [32]. Even though little is known about it, some studies provide strong evidence that modulation of the hem- and lymphangiogenic response critically modulates the immune response and vice versa [33,34,35]. They furthermore indicate that modulation of the immunological microenvironment, such as through induction of tolerogenic mechanisms, can improve corneal transplant survival despite the presence of blood and lymphatic vessels.

Transplantation of allogeneic tissue always comes with the risk of rejection and the loss of the graft due to immune responses against the introduced tissue. This is also true for corneal transplants. Prolongation of transplant survival and in best case, lifelong acceptance of the transplant, is a big challenge for research groups around the world, including us. The establishment of a tolerogenic environment would be a highly requested approach in the transplantation setting. Contrary to immunosuppression, the induction of immune tolerance would preserve the ability of the recipient to manage pathogenic conditions independent of the transplantation. In the following sections, this review will give an overview on the different strategies to improve the outcome of corneal transplantation. Due to their significance in the field, approaches targeting (neo) vascularization are described too. Thereafter, the focus is on strategies promoting tolerogenic mechanisms by targeting DCs.

## 2. Antiangiogenic Strategies

Since the cornea’s vascularization is considered as a major risk factor for transplant survival on the one hand, and is deeply involved in the immunological response to the graft on the other, this field takes up a significant part of corneal transplant research.

The application of recombinant antibodies or antibody fragments, such as Ranibizumab, vascular endothelial growth factor receptor (VEGF-R) 1/2 trap [36], VEGF-R3 antibody [30,37], vascular endothelial growth factor (VEGF)-C/D trap [38], targeting VEGF-A, -C, and -D or their receptors VEGF-R1, -R2, or -R3, is showing promising results regarding the inhibition of corneal hem- and lymphangiogenesis as well as the improvement of corneal transplantation [32]. Furthermore, the blockade of insulin receptor substrate 1(IRS-1) by the 25mer DNA antisense oligonucleotide named Aganirsen inhibits vessel ingrowth into the cornea through an anti-proliferative effect on LECs and decreased VEGF-A expression levels [39]. The topical application of Aganirsen in a Phase III study confirmed its effects on neovascularization in patients with keratitis and furthermore reduced the need for transplantation [40]. In addition to members of the VEGF family, vasohibin-1 [41], endostatin [42], semaphorin 3F (sema3f) [43], and recently tyrosinase [44] were also identified as modulators of the lymphangiogenic response. Notably, the topical application of sema3F significantly improved graft survival in a high-risk model of murine corneal transplantation [43]. However, the effect of these strategies on the immune system is only a little or not understood so far.

In a recent study, we focused on the underlying mechanisms of anti-angiogenic treatment in corneal transplantation and revealed that the immunological microenvironment plays a critical role. VEGF-A depletion by topical application of a VEGF-R1/R2 trap improved graft survival in a murine model of high-risk corneal transplantation by altering the chemokine and cytokine profiles, inhibiting CD11c+ DC infiltration into the cornea, as well as by upregulation of the tolerance-inducing marker forkhead transcription factor Foxp3 [33]. Another study showed that blocking of the chemokine (C-C motif) receptor 7 (CCR7) also prolonged corneal transplant survival in pre-vascularized recipients by reducing immune cell trafficking [35]. CCR7 is usually upregulated by DCs upon activating stimuli and initiates their migration to lymphatic vessels that express the chemokine CCL21 [29]. As described in the introduction, migration of DCs to the LNs is necessary for their interaction with T cells and the induction of an adaptive immune response. Interestingly, a study by Hos et al. indicated that lymphatic vessels promote corneal resolution of inflammation [34]. They revealed that IL-10 exerts anti-inflammatory properties in inflamed corneas by promoting lymphangiogenesis indirectly via macrophages. In accordance with that, IL-10-deficiency was associated with reduced lymphangiogenesis and more severe inflammatory responses [34]. In summary, the latter studies describe an interdependence between blood and lymphatic vessels and immune cells, including their cell signaling molecules. Moreover, they not only highlight the importance of the immunological microenvironment, but also indicate the great potential of strategies that render this microenvironment tolerogenic and thus improve the outcome of corneal transplantations. In the following sections, these strategies are described with a focus on DCs, since they are considered a central player in the immune system.

## 3. Soluble CD83 (sCD83)

CD83 serves, next to other co-stimulatory molecules like CD40, CD80, and CD86, as a phenotypical marker for mature DCs [45,46]. The molecule belongs to the immunoglobulin (Ig) superfamily, with an extracellular Ig-like domain, a transmembrane domain, and an intracellular domain. However, its functional significance has long been unknown [46,47]. About two decades ago, Kruse et al. revealed that the inhibition of CD83 protein synthesis abolished the capacity of DCs to stimulate T cells [46].

On the other hand, in vitro experiments revealed that incubation of immature DCs with a recombinant protein containing the extracellular domain of human CD83 (hrCD83ext) inhibited their maturation, as CD80 and CD83 expression itself were downregulated [48,49]. Moreover, their capacity to stimulate allogeneic T cells was significantly reduced [48,49]. These results demonstrated immune-regulatory functions of the extracellular domain of CD83 similar to tolDCs. Interestingly, a soluble form of CD83 (sCD83) was identified, which is predominantly released from activated DCs and B lymphocytes and detectable in human sera from healthy donors [49]. Further studies investigated the immune-regulatory function of sCD83 and reported among others a protective effect in experimental autoimmune encephalomyelitis as well as in experimental autoimmune uveitis [50,51] and a preventive effect in inflammatory bowel disease [52]. It was associated with a reduction in cytokine levels of IFN-γ, IL-2, and IL-4 as well as long-term IDO expression by DCs [50,51,52]. Application of sCD83 also showed therapeutic potential in corneal transplantation.

Systemic application of sCD83 in a murine high-risk transplantation model significantly prolonged corneal graft survival. It was associated with a significantly increased frequency of Foxp3+ Tregs in the dLNs and an increased expression of FoxP3 and IDO in the cornea [53].

**Figure 1:**
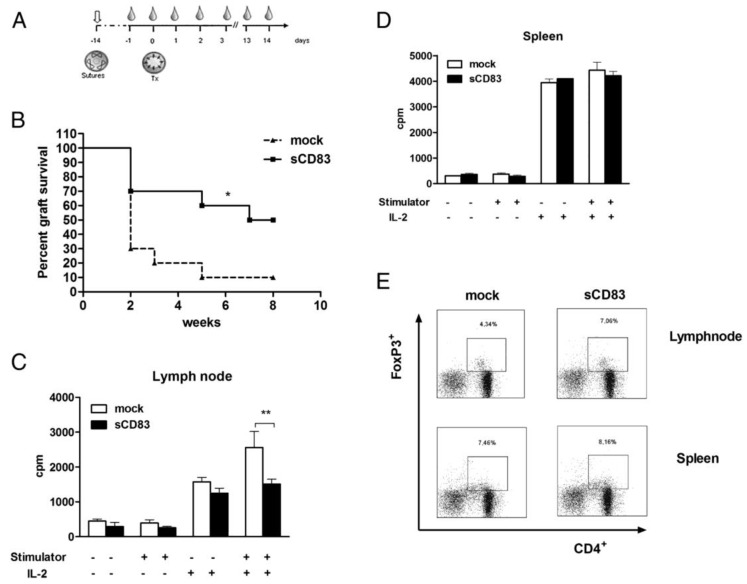
Topical application of sCD83 induces allogeneic graft tolerance, reduces proliferative response to donor alloantigen, and induces Foxp3+ regulatory T cells. (**A**) Experimental design and timescale of sCD83 administration. Sutures were placed in the cornea to induce inflammation (sutures) 14 d prior to transplantation (Tx). sCD83 (7.5 mg per 3 mL) was applied in the form of eye drops three times per day on indicated time points (drops). (**B**) The topical sCD83-treated group had a significantly improved graft survival compared with the mock-treated group (*p* < 0.025; number of animals in each group: *n* = 10). * *p* < 0.05. (**C**) Draining lymph node cells from sCD83-treated animals showed a significantly reduced proliferation capacity in the presence of alloantigen and IL-2 compared with the mock-treated animals. Cells derived from sCD83-treated animals were still able to respond to exogenous IL-2, but to a lesser extent than mock-treated mice. Data are presented as the mean ± SE of triplicate samples. (**D**) This effect could not be detected in restimulated splenocytes, neither in the sCD83- nor in the mock-treated group. (**E**) Single-cell suspensions of neck lymph nodes and spleens derived from different treatment groups were analyzed by FACS and gated on CD3+ lymphocytes. The proportion of CD4+ T cells expressing Foxp3 in the lymph node was increased in sCD83-treated animals in comparison with the mock-treated group (upper panel). Again, this effect could not be observed in the spleen (lower panel). Representative FACS plots are shown. The experiment represents the data of cells from five pooled mice. ** *p* < 0.01 [53].

Since treatment with eye drops is easier and more favorable in terms of clinical application, the topical way of administration was also investigated in corneal transplantation. It induced local, allogeneic graft tolerance in murine high-risk recipients and reduced the proliferative response of T cells from LNs but not from the spleen to donor alloantigen. T cells were not driven into apoptosis or anergy, but instead developed a tolerogenic phenotype, since Foxp3+ T cell frequency was increased in the LNs (Figure 1). Furthermore, topical sCD83 administration led to higher expression of IDO and TGF-β in the eye dLNs. Blocking of either IDO or TGF-β abrogated the positive sCD83-mediated effect on graft survival, proving that both play a major role in this context and pointing towards the activation of a cascade that induces long-term tolerance [53].

IDO is a rate-limiting enzyme in tryptophan metabolism, which is expressed among others by DCs [54,55,56]. In naïve corneas, IDO is expressed at low levels, but the expression is strongly upregulated upon pro-inflammatory stimulation with IFN-γ and/or TNF-α [57]. This axis is effective in managing acute immune responses, whereas stimulation with TGF-β was observed to induce long-term IDO activation by establishing a tolerogenic microenvironment [55,58]. It includes the shift of T lymphocytes towards a tolerogenic phenotype due to tryptophan starvation combined with the production of tryptophan catabolites, referred to as kynurenines [59,60,61]. The long-term effect is achieved by the process of “infectious tolerance”, which describes the capacity of tolerogenic lymphocytes to induce and thus spread the tolerance state in other cell types by the production of soluble factors, such as kynurenines, TGF-β, as well as type I and type II interferons [58,62].

The importance of IDO in corneal transplantation has been strengthened as its overexpression in murine corneas [57] as well as the topical and systemic application of the tryptophan catabolite, 3-hydroxykynurenine, and the tryptophan metabolite analogue, N(-3,4-dimethoxycinnamonyl) anthranilic acid, prolonged allograft survival [63,64].

## 4. Co-Stimulatory Molecules

As already mentioned, antigen-specific T cell signaling requires not only the interaction of the TCR with MHC molecules on APCs but also ligation of co-stimulatory molecules [65]. Best characterized are the interactions between CD80 and/or CD86 on APCs with CD28 or CTLA4 on T cells as well as CD40 with CD154, respectively. Further receptors for co-stimulatory molecules were characterized more recently, including inducible co-stimulatory molecule (ICOS) and programmed death molecule (PD) 1 and 2 binding to their own ligands on APCs ICOS-L and PD-L, respectively [23,66]. However, activation of the TCR without co-stimulatory interactions results in depletion of activated T cells, T cell anergy, or clonal unresponsiveness. These pathways, therefore, display a promising target in terms of modulating the immune response to promote engraftment [22,23].

### 4.1. Cytotoxic T Lymphocyte-Associated Antigen 4 (CTLA4)

CTLA4 is a transmembrane protein that binds to CD80 and CD86 on APCs [22]. CTLA4 becomes upregulated on naïve T cells upon activation, whereas it is constitutively expressed on Tregs [22,67]. It shares its receptors with CD28 but both binding avidity and affinity of CTLA4 are significantly higher, which is why it is considered as a competitive antagonist [22]. Its importance as negative regulator of T cell responses is undisputed, as CTLA4-knockout mice develop a fatal lymphoproliferative disorder [68]. However, the precise mechanism of action of CTLA is not fully unravelled. Qureshi et al. revealed that CTLA4 removes its co-stimulatory ligands CD80 and CD86 from APCs via trans-endocytosis in vitro as well as in vivo, thereby preventing the ligation of CD28 known to efficiently promote T cell survival and proliferation [67,68]. Moreover, CTLA4 engagement was reported to induce IDO production in APCs, consequently promoting Treg function [65]. Thus, modulation of this signalling axis seemed to be promising in the transplantation setting.

An already investigated approach in cornea transplantation is the application of CTLA4 or CTLA4-Ig, a recombinant fusion protein containing the extracellular domain of human CTLA4 fused to the human IgG1 constant region [69]. Several ways of administration have been analysed. Local treatment, including subconjunctival injection, ex vivo viral-mediated gene transfer or graft pre-incubation with CTLA4-Ig protein showed modest, unsatisfying effects [69,70,71,72]. Systemic treatment by intraperitoneal injection or adenovirus (Ad)-mediated gene transfer of CTLA4-Ig revealed the most promising results regarding the prolongation of allograft survival [69,71,72]. The use of CTLA4-Ig in corneal transplantation, contrary to other transplantation models, has a positive effect on acute allograft rejection than contributing to long-term allograft survival [73,74,75,76,77,78].

Another combination analysed in corneal transplantation was CTLA4 fused to Fas ligand (CTLA4-FasL). FasL ligation to its receptor Fas results in apoptosis of the receptor-bearing cells [79]. Fas expression is not specific for any cell type, whereas FasL is predominantly expressed on immune cells, including monocytes, NK cells, and activated B and T cells, as well as on some cells in the testis and the eye [79,80]. The axis not only plays a crucial role in maintaining the cornea’s immune privilege but also contributes significantly to the success of corneal transplantation. Corneal grafts transplanted into FasL deficient mice were rejected 100% [81]. To include FasL in the research of therapeutics prolonging corneal graft survival is therefore obvious. However, the effectivity of FasL is reduced by increased co-stimulation via the CD28-CD80/CD86 axis [82,83]. Vice versa, blockade of this pathway is stimulatory on Fas-mediated apoptosis [84,85]. Consequently, CTLA4-FasL fusion protein combines two factors, which are in favor of prolonging graft survival. Moreover, the fused variant was more effective than CTLA4-Ig or soluble FasL alone or combined [80]. When murine corneal grafts were pre-incubated with CTLA4-FasL, survival rates exceeded three months and this was associated with reduced CD4+ T cell frequencies and induction of apoptosis in predominantly infiltrating cells [86]. It is conclusive since acute rejection is mediated primarily by CD4+ T cells, which are more susceptible to Fas-mediated apoptosis compared to CD8+ T cells [87,88,89]. Both, long-term allograft survival as well as corneal engraftment being unaffected by the introduction of skin transplants point towards donor-specific tolerance following CTLA4-FasL treatment [86]. However, conclusive findings for an established tolerogenic environment, such as increased Treg frequencies or upregulation of IDO, were not shown.

### 4.2. CD40–CD154

The interaction between CD40 and CD154 is another well-characterized co-stimulatory pathway. CD40 is expressed on several cell types, including DCs, B cells, and macrophages [90]. Stimulation of CD40 by TNF family members, including CD154, induces DC maturation by upregulation of MHC molecules as well as co-stimulatory molecules on the cell surface [91]. CD154 expression is upregulated on T cells upon stimulation. Furthermore, it is expressed on monocytes, platelets, and B cells [90]. Engagement of CD154 with CD40 stimulates upregulation of CD80 and CD86 on DCs and thereby promotes CD28-mediated T cell activation. Furthermore, the interaction stimulates the release of pro-inflammatory cytokines and chemokines, including IL-12, IL-8, MIP-1α and β, and leads to an upregulation of adhesion molecules, such as intracellular adhesion molecule 1 (ICAM1) [11,90].

Blockade of the pathway initially showed promising results in several murine transplant models [92,93,94,95,96]. The application of an anti-CD154 antibody limited APC maturation and consequently down-modulated the interaction of CD28 with CD80 and CD86. This resulted in the lack of the second signal for T cell activation and in the end elicited T cell anergy [97]. Regarding corneal transplantation, intraperitoneal injection prevented at least acute allograft rejection, associated with a reduction in infiltrating immune cells into the graft and LNs, as well as a decrease in IFN-γ producing T cells [98,99]. However, as soon as the treatment was stopped, the transplants were rejected [98]. This was also true for the local, subconjunctival application of the antibody, where acute graft rejection was anticipated by dampening the pro-inflammatory Th1 response and infiltration of leukocytes to the graft site [98,100]. Although anti-CD154 application suppressed harmful reaction, it failed to induce profound tolerogenic processes and thus long-term corneal graft-survival [98,99,100,101].

### 4.3. Inducible T Cell Co-Stimulator (ICOS)

More recently, another member of the CD28 family has been identified. ICOS is structurally related to CD28 and CTLA4, but does interact neither with CD80 nor with CD86 on DCs. It is expressed on both CD4+ and CD8+ T cells after CD28 signalling and binds to ICOSL, which is constitutively expressed at low levels on B cells, macrophages, and DCs [102,103,104]. Inflammatory stimuli induce the upregulation of ICOSL on APCs and the expression in non-lymphoid tissues such as heart, lung, kidney, and testes [104]. ICOS-ICOSL interaction plays a significant role in T cell activation and differentiation to Th1 or Th2 cells, respectively, as well as in tailoring the immune response through cytokine production [68,105,106]. While the impact on T cell function is intensively investigated, and reveals complex interactions and dependencies, only few studies have focussed on the effect on DCs [107]. However, studies performed in mice showed an immunogenic effect of ICOSL, triggering partial maturation of iDCs and increased secretion of IL-6 [107]. On the contrary, experiments performed on human DCs revealed no differences of ICOSL stimulation on the expression of co-stimulatory molecules [108]. ICOSL triggering was even associated with increased IL-10 secretion as well as inhibited adhesion of both iDCs and mDCs to vascular and lymphoid endothelial cells and down-modulated migratory activity [108]. Even though the studies showed contrary results, probably due to species differences, the latter findings particularly indicate that modulation of this axis could be beneficial in (corneal) transplantation.

In a cardiac transplantation model, ICOS expression was shown to be upregulated during the establishment of allograft rejection [102]. Moreover, blockade of the pathway was beneficial for graft survival in several transplantation models, including heart, liver, and islet cells [102,103,109]. On the contrary, the systemic application of a blocking antibody in corneal transplantation had no effect on graft survival [66].

However, ICOS seems to be important for tolerance homeostasis. ICOS-deficient mice showed difficulties in tolerance induction, which was accompanied by reduced Treg frequencies [110,111,112,113]. Furthermore, ICOS functioned as an indicator for both survival and potency of Tregs since ICOS-deficient Tregs possessed less suppressive activity and were more susceptible to die [114]. In the model of corneal transplantation, allograft rejection was associated with abrogated ICOSL expression accompanied by infiltrating ICOS+ Foxp3- CD4+ T cells into the graft. Moreover, both transplantation of a graft into ICOS deficient mice as well as into WT mice treated intraperitoneally with an anti-ICOSL mAb shortened graft survival [115]. Thus, contrary to other transplantation models, the ICOS-ICOSL pathway is apparently required for graft survival of the cornea, suggesting that its administration or overexpression may be beneficial.

Unfortunately, neither ex vivo gene transfer of an ICOS-Ig nor systemic gene therapy had an effect on corneal graft survival in rats [116]. Even though the application of ICOS has not been shown to have a positive effect on the outcome of corneal transplantation on its own, its combination with other compounds showed more promising results. This is discussed at the end of this chapter.

### 4.4. Programmed Death Protein 1 (PD-1)–PD-L1

As ICOS, PD-1 is a structural homologue to CD28 and CTLA4. It is expressed on activated T cells, B cells, and myeloid cells [117]. Ligands identified so far are PD-L1 and PD-L2. PD-L1 is constitutively expressed on several non-hematopoietic cells as well as on T cells, B cells, macrophages, and DCs on which its expression is further upregulated upon activation [118,119]. PD-L2 is inducibly expressed primarily on DCs and macrophages [119]. Notably, DCs express both PD-1 receptor as well as its ligands and thereby are not only capable to interact with any PD-1 and PD-L1 positive cell but once more demonstrate their importance as orchestrators of the immune system [120]. However, the best investigated interaction is between DCs and T cells.

Functional analyses of PD-1 using different mouse knockout strains revealed immune inhibitory properties as knockout mice developed pathogenic conditions such as lupus-like arthritis or chronic GvHD with a breakdown of peripheral tolerance [117,121]. Vice versa, PD-1 activation downregulated immune responses by inhibiting lymphocyte proliferation and contributed to peripheral tolerance by promoting induced Treg development and maintenance [117,119]. Kuipers et al. investigated the role of PD-L1 and PD-L2 in CD4+ T cell activation [122]. Overexpression of these molecules in DCs led to decreased CD4+ T cell activation in DC-T cell co-cultures. Surprisingly, the addition of soluble PD-1 (sPD-1) showed a similar effect, which the authors attributed to the phenomenon of reverse signalling. Further analyses of DCs stimulated with sPD-1 revealed a tolerogenic phenotype with decreased expression of CD40, CD80, and CD86 and elevated production of IL-10 [122].

Regarding the cornea, different studies showed that PD-L1 is constitutively expressed on corneal endothelial, stromal, as well as epithelial cells and is upregulated at least on epithelial cells when inflammatory stimuli were introduced [123,124]. It was suggested that PD-L1 contributes to the immune privilege, since infiltrating T cells into the cornea underwent apoptosis. This effect was abrogated when PD-L1-PD-1 interaction was blocked. Furthermore, systemic treatment of the recipient with PD-L1- or PD-1-blocking antibodies increased the rejection rates of corneal allografts [123]. The contribution of PD-L1 to the privileged status of the cornea was further characterized. The loss of PD-L1 was associated with a more profound angiogenic response and increased VEGF-R2 expression after introducing an angiogenic stimulus by suture-placement [125]. As these studies identified PD-L1 as an important factor for the privileged status of the cornea, it was obvious to test its therapeutic potential on prolonging corneal allograft survival. Systemic application of a PD-L1 Ig fusion protein, consisting of the extracellular PD-L1 domain fused to the Fc portion of human IgG, resulted in prolonged corneal allograft survival. The authors suggested an additive effect of endogenous PD-L1 expressed in the cornea and systemically applied PD-L1 Ig acting in the LNs [66]. Local overexpression of PD-L1 in corneal grafts mediated by lentiviral transduction resulted in significantly longer transplant survival and was associated with a reduction in graft-infiltrating cells [126]. Topical application of PD-L1 in the form of eye drops has, to our knowledge, not been investigated so far. Altogether, PD-L1 plays a critical role in the corneal transplantation setting, as its constitutive expression seems to contribute to the cornea’s privileged status and its application shows therapeutic potential. As an interim summary, targeting the second, co-stimulatory signal required for T cell activation is a promising approach in the field of transplantation in general. Regarding corneal transplantation, only PD-L1 and CTLA4-FasL revealed satisfying effects when applied alone.

Strikingly, the outcome of all strategies on DCs is similar even though reliable data may not exist for all of them due to the investigational focus on T cells. However, it is obvious that inhibition or stimulation, depending on the nature of the respective pathway, of the second signal of T cell activation decreases the expression of co-stimulatory molecules, especially of CD80 and CD86. Moderate expression of co-stimulatory molecules is characteristic for tolDCs. This is also true for increased IDO expression that is associated with CTLA4 ligation to CD80 and CD86, decreased production of IL-12, which is a result of the blockade of CD40-CD154 interaction, as well as increased levels of IL-10 described as a consequence of ICOSL and PD-L1/-L2 stimulation. Moreover, ICSOL stimulation has been shown to inhibit the adhesion of DCs to lymphatic endothelial cells (LECS) as well as to decrease their migratory activity. This is of significance, especially for corneal transplantation, where immune cell trafficking through blood and lymphatic vessels play a superior role. Altogether, it indicates that modulating the interaction between DCs and T cells by targeting co-stimulatory molecules contributes to the establishment of a tolerogenic microenvironment.

In some transplant models, the combination of the above described agents has been shown to be successful and might represent an alternative therapeutic strategy [22,92,93,95,127,128]. Therefore, future approaches might focus on a more combined modulation of these different pathways.

## 5. Thrombospondin-1 (TSP-1)

TSP-1 is a molecule with an already approved potential to promote long-term corneal allograft survival through the establishment of a tolerogenic environment. It belongs to a family of five extracellular matrix proteins and is predominantly secreted by platelets, monocytes, and macrophages [129,130]. Because of its ligation to several surface receptors, including CD36 and CD47 on DCs, and interaction with a variety of cytokines and growth factors, TSP-1 plays a role in numerous cell functions [129,131]. However, several observations led to the conclusion that TSP-1 possesses potent anti-inflammatory properties [129,132,133,134]. TSP-1-deficient mice develop a severe inflammatory phenotype. TSP-1 is secreted by apoptotic monocytes and in general expressed at high concentration in damaged and inflamed tissue [129,132,133,134]. Moreover, it was identified as a major activator of TGF-β, which potently induces immune regulatory mechanisms such as long-term IDO activation as well as Treg generation by stimulating FoxP3 expression [55,58,133,135].

Notably, a prerequisite for TGF-β activation by TSP-1 is the tethering of TSP-1 to APCs via its receptor CD36 [136]. In addition to its indirect anti-inflammatory effect exerted through TGF-β activation, TSP-1 also directly affects DCs [137]. Binding of TSP-1 to CD47 on mDCs suppresses their production of the pro-inflammatory cytokines IL-12, TNF-α, IL-6, and GM-CSF. Ligation of TSP-1 to iDCs prevents both phenotypical and functional changes characteristic for mature DC. This includes the upregulation of MHCII and co-stimulatory molecules as well as the capability to potently stimulate T cells [137].

TSP-1 is constitutively expressed in healthy murine eyes and known as a regulator of the local APC phenotype maintaining ocular surface health as well as of anti-angiogenic factors contributing to the cornea’s angiogenic privilege [7,59]. Six month-old TSP-1-deficient mice developed increased spontaneous corneal lymphangiogenesis [7,138]. In young TSP-1-deficient mice, inflammation-induced corneal neovascularization could be reversed by the topical application of recombinant human TSP-1 [138]. Since neovascularization is acknowledged as a high-risk factor for the success of corneal transplantation, these data indicate an essential role of TSP-1 in the transplantation setting. This was supported by Saban et al., who transplanted TSP-1 null allografts into wild type mice and observed an increase in rejection rates from 50% to nearly 100% when TSP-1 was missing. Surprisingly, analysis of the underlying pathway of allorecognition revealed that the direct pathway and, not as expected, the indirect pathway was dominant. Furthermore, TSP-1-deficient APCs showed a more mature phenotype and acquired a more potent T cell stimulating activity [139].

Interestingly, CCR7 expression was upregulated in TSP-1 deficient mice whose blockade has been shown to be effective in promoting low-risk corneal graft survival [35,139]. Conversely, these results indicate that TSP-1 could exert profound anti-inflammatory effects when applied systemically or topically after corneal transplantation. Even though no studies are available for corneal transplantation, Soriano-Romani et al. showed that a peptide derived from TSP-1 (KRFK) can activate TGF-β, exert its reducing effect on co-stimulatory molecule expression on APCs, and prevent the development of chronic ocular inflammation in TSP-1 deficient mice [140]. The role of TSP-1 as a therapeutic improving the high-risk and long-term outcome of corneal transplantation is encouraging.

## 6. Interleukin-10 (IL-10)

Solely in this review has TGF-β appeared in different sections, highlighting its complex and critical role in transplantation as an immune-modulatory cytokine. Therefore, defining the importance of IL-10 is inevitable. As an immune-modulatory cytokine, IL-10 is produced by various cell types, including activated T cells, B cells, keratinocytes, macrophages, and DCs [141,142]. Amongst other properties, IL-10 can directly render DCs tolerogenic and thus inhibit their T cell stimulatory capacity or even promote T cell anergy-eliciting mechanisms [142,143]. However, disregarding the prominent anti-inflammatory properties of IL-10, its effectivity in corneal transplantation models is marginal. Subconjunctival injection showed no beneficial effect and gene therapy revealed inconclusive results [144,145]. In the sheep model, transfection of donor corneas with IL-10 cDNA reduced corneal rejection rates, whereas in rats neither liposomal nor adenoviral graft transfection improved graft survival [145]. Only systemic application gene transferred IL-10 showed beneficial effects [145].

However, Tahvildari et al. analysed the combined application of the two immune-modulatory cytokines TGF-β and IL10 in the model of murine corneal transplantation. When donor-type bone marrow dendritic cells (BMDCs) were treated with a combination of IL-10 and TGF-β1, they developed a tolerogenic phenotype. Systemic transfer of those tolDCs to corneal transplant recipients significantly improved allograft survival [13]. In a subsequent study, the group injected the combination of immune-modulatory cytokines subconjunctivally to donor animals, which rendered the corneal microenvironment tolerogenic. Subsequent transplantation of these “engineered” grafts prolonged corneal graft survival and was associated with abrogated IFN-γ secreting CD4+ T cells [146]. Even though subconjunctival injection of therapeutics into the donor is not translatable into the clinic, the study showed that manipulation of the donor tissue and its cells and thus, once more, the direct pathway of allorecognition indeed play an important role in transplantations. Since the cornea is stored for up to four weeks in eye banks, therapeutic treatment prior to transplantation is a strategy worth considering.

## 7. Activated Leukocyte Cell Adhesion Molecule (ALCAM)

As already mentioned, pre-vascularized corneal graft beds have a high-risk status since immune cell trafficking is facilitated by both blood and lymphatic vessels. Proteins that further promote leukocyte recruitment and activation as well as their interaction with (blood) ECs are cell adhesion molecules (CAMs) [147,148,149]. One of them is ALCAM, a member of the Ig superfamily that is expressed by several cell types, including activated T cells, monocytes, as well as DCs, fibroblasts, ECs, and keratinocytes [150,151]. Expressed on ECs, ALCAM has been identified to play a major role in leukocyte trafficking, T cell activation, and vascular development [148,152,153,154]. Compared to blood vessels, leukocyte trafficking through lymphatic vessels is much less characterized [29]. However, they constitute the means of transport for DCs to dLNs, where they induce adaptive immune responses [29]. Adhesion molecules have been shown to be essential for lymphatic vessel development and function, which is also true for ALCAM [152]. An ALCAM-dependency has been reported for LEC migration, tube formation, as well as LEC-LEC interactions. Focusing on DCs, blockade of ALCAM reduced the adhesion of DCs to LEC monolayers [152]. Studies on in vitro-cultured human skin biopsies showed reduced emigration from DCs when ALCAM was blocked [155]. However, its confirmed role in the organization and function of lymphatic vessels, together with an indicated effect on DC migration, suggests therapeutic potential.

In corneal transplantation, the application of an ALCAM-blocking antibody would not only target the (in)growth of harmful vessels into the cornea, but also the immune cells trafficking through them. The systemic application of a recently developed antibody blocking murine ALCAM effectively prevented corneal allograft rejection in a mouse model of high-risk corneal transplantation. Compared to the control-treated mice, Treg frequencies in dLNs were significantly increased, indicating tolerance-inducing conditions [155]. Surprisingly, the beneficial effect of ALCAM blockade on corneal transplant survival could not be attributed to impaired lymph- or hemangiogenesis, since no difference was detected in vessel ingrowth into the cornea between treated and control mice. However, the investigators observed higher numbers of CD11c+ cells in the cornea. They showed in an additional transplantation experiment that blockade of ALCAM prevented the emigration of DCs from the cornea to the dLNs, which was associated with decreased T cell numbers in the cornea. They concluded that the blockade of ALCAM promotes corneal allograft survival by reducing DC emigration to the dLNs, thus preventing the induction of an adaptive immune response. Together with another study, which showed that blockade of the CCL21/CCR7 axis [35] and therefore mDC migration favors corneal engraftment, this data uncovered new therapeutic targets in the transplantation setting.

## 8. Adoptive Cell Transfer

Another innovative strategy in the field of (corneal) transplantation aiming at long-term engraftment is “negative vaccination” with tolDCs. Since DC vaccines are successfully used in prostate cancer treatment, it is worth considering the efficacy of tolerogenic cell therapy in transplantations [156]. Safe application of autologous tolDCs has been demonstrated in patients with Type I diabetes and has been tested in kidney transplant recipients [156,157]. Zhou et al. reviewed studies focusing on the adoptive transfer of tolDCs on allograft survival in different animal organ transplantation models [156]. Although the outcome was dependent on the protocol for the ex vivo generation of tolDCs, the animal and transplantation model, as well as the animal strain, the method showed great potential [156]. In murine corneal transplantation, intravenous (IV) injection of donor-derived tolDCs significantly prolonged corneal allograft survival, accompanied by decreased IFN-γ+ T cell frequencies and increased Foxp3 expression in dLNs [13]. Furthermore, Yan et al. reported that IV administration of syngeneic tolDCs pulsed with donor antigen also resulted in an improved corneal transplantation outcome [158]. Although the injection of donor cells prior to transplantation is challenging to transfer into the clinic with regards to host sensitization, the studies indeed proved the method’s potential. Notably, the group of Morelli showed that injection of unpulsed recipient tolDCs is able to induce tolerance in heart transplantation [159].

## 9. Conclusions

**Figure 2:**
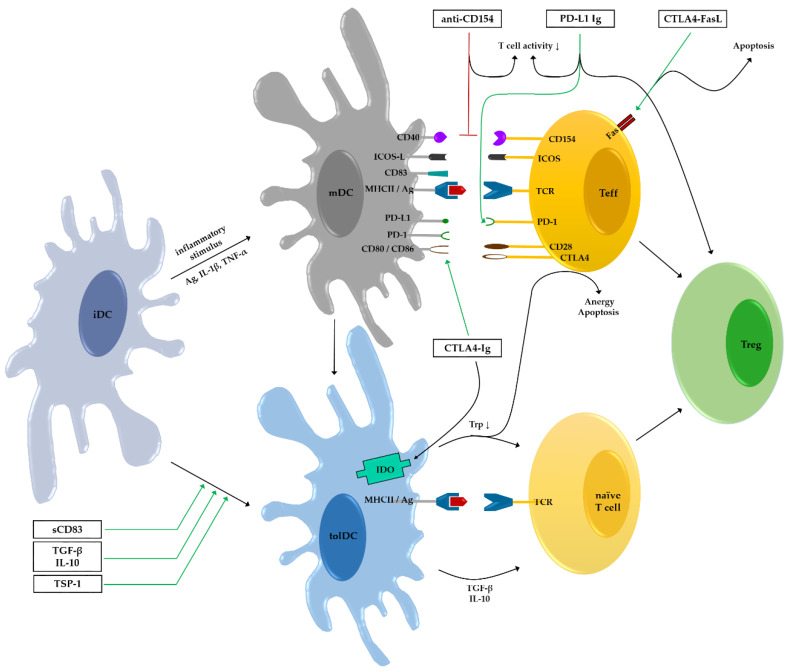
Overview of immune modulatory strategies to improve corneal graft survival targeting dendritic cells (DCs). Stimulatory effects of compounds are depicted in green, inhibitory effects of compounds are depicted in red. Ag–antigen; iDC–immature DC; mDC–mature DC; Teff–effector T cell; tolDC–tolerogenic DC; Treg–regulatory T cell; Trp–tryptophan.

DCs are known as central mediators and connectors of the adaptive and innate immune system. Their ability to induce tolerogenic mechanisms promoting engraftment of (corneal) allografts can be used to an advantage. Various strategies can be pursued, including direct stimulation of DC differentiation to tolDCs, modulation of DC-T cell interactions that result in T cell anergy or Treg generation, and influencing the migration capacities of DCs (summarized in Figure 2). However, some approaches were only successful or only investigated when the compound was applied systemically (Table 1). This should be done with care since this way of administration is often accompanied by severe side-effects compared to local treatment options such as eye drops. Unfortunately, none of the described molecules has made it to clinical trials so far and further studies are required to determine if compounds that improve engraftment when applied systemically are also efficient as topically applied therapeutics or translatable into human corneal transplantation. To further limit side-effects, manipulation of the donor tissue is worth further research. Ultimately, from all the described strategies, a combined treatment might have a better effect on the outcome of corneal graft survival.

## Figures and Tables

**Table 1:** Summary of immunomodulatory therapies to prevent corneal graft rejection targeting dendritic cells.

Compound	Function/Effect	Treatment	Results	Ref.
sCD83	tolerogenic	systemic	prolongation of corneal graft survival*	[52]
		eye drops	prolongation of corneal graft survival*	[52]
CTLA4Ig	mimics CTLA4-CD80/CD86 interaction	systemic	moderate effect on corneal graft survival*^†^	[71,73]
		subconjunctival	no effect on corneal graft survival*	[71]
		pre-incubation	prolongation of corneal graft survival^†‡^	[72,73]
		ex vivo gene transfer (viral)	moderate effect on corneal graft survival^†^	[73,74]
		systemic gene transfer (viral)	prolongation of corneal graft survival^†^	[73,74]
CTLA4-FasL	mimics CTLA-CD80/CD86 interaction facilitating Fas-mediated apoptosis	pre-incubation	prolongation of corneal graft survival*	[87]
anti-CD154	blocks CD40-CD154 interaction	systemic	prolongation of corneal graft survival*^1^	[97,98]
		subconjunctival	prolongation of corneal graft survival*^1^	[99]
anti-ICOS	blocks ICOS-ICOSL interaction	systemic	no effect on corneal graft survival*	[69]
ICOS-Ig	mimics ICOS-ICOSL interaction	ex vivo and systemic gene transfer (viral)	no effect on corneal graft survival^†^	[113]
PD-L1	stimulates PD-1	ex vivo gene transfer (viral)	prolongation of corneal graft survival^†^	[123]
PD-L1-Ig	mimics PD-L1-PD-1 interaction	systemic	prolongation of corneal graft survival*	[69]
IL-10	inhibits Th1 immune response, induces tolDCs	subconjunctival	no effect on corneal graft survival^†^	[139]
		systemic	no effect on corneal graft survival^†^	[139]
		ex vivo gene transfer (viral)	prolongation of corneal graft survival^Φ^	[140]
		ex vivo gene transfer (plasmid/liposome)	moderate effect on corneal graft survival^†^	[141]
		systemic gene transfer (viral)	prolongation of corneal graft survival^†^	[141]
IL-10, TGF-β	inhibits Th1 immune response, induces tolDCs	local treatment of the donor	prolongation of corneal graft survival*	[142]
anti-ALCAM	blocks ALCAM	systemic	prolongation of corneal graft survival*	[151]

^1^ until therapy’s discontinuation; *—in mice; †—in rats; ‡—in rabbits; Φ—in sheep.
